# Association between Copeptin and Metabolic Syndrome: A Systematic Review

**DOI:** 10.1155/2022/5237903

**Published:** 2022-10-22

**Authors:** Ricardo Rojas-Humpire, David R. Soriano-Moreno, Brenda Galindo-Yllu, Jessica Hanae Zafra-Tanaka

**Affiliations:** ^1^Unidad de Investigación Clínica y Epidemiológica, Escuela de Medicina, Universidad Peruana Unión, Lima, Peru; ^2^Grupo de Investigación P53, Escuela de Medicina Humana, Universidad Peruana Unión, Lima, Peru; ^3^Escuela de Medicina, Universidad Científica del Sur, Lima, Peru

## Abstract

**Background:**

Copeptin, a reliable marker for vasopressin release, has been associated with cardiometabolic diseases including metabolic syndrome (MetS). This systematic review aims to evaluate the association between copeptin and MetS.

**Methods:**

We searched in Pubmed, Scopus, EMBASE, and Web of Science databases until March 2021 and included observational studies (cohort studies, cross-sectional, and case-control) reporting the risk or prevalence of having MetS in patients with elevated copeptin levels compared to patients without elevated copeptin levels. The risk of bias was evaluated with the Newcastle-Ottawa Scale. Meta-analysis was not performed because of the heterogeneity of the copeptin cut-off values.

**Results:**

A total of 7 studies (5 cross-sectional, 1 case-control, and 1 cohort) were included comprising 11,699 participants. Most of them were performed in the adult general population. Two cross-sectional and one case-control studies found a positive significant association between higher levels of copeptin and MetS. While three cross-sectional and one cohort studies found no association. The case-control study had several methodological limitations, most cross-sectional studies were methodologically adequate and the cohort study had no methodological issues.

**Conclusions:**

The association between copeptin and MetS is inconsistent. However, the arginine-vasopressin system impairment contributes to metabolic disorders, expressing plasma copeptin changes. Thus, more longitudinal studies are required to corroborate the association of copeptin and MetS.

## 1. Introduction

Metabolic syndrome (MetS) is a set of interrelated disorders characterized by hypertension, hyperglycemia, obesity, and insulin resistance [1]. This pathologic condition is very common around the world, the estimated worldwide prevalence is 25%, approximately three times more frequent than type 2 diabetes mellitus (T2DM). However, the frequency and distribution of MetS vary according to the diagnostic criteria applied, race, culture, and geographic location [2–4].

The impact of MetS is evident as a risk factor for T2DM, atheroma plaque formation, acute myocardial infarction, cerebrovascular disease, and other cardiovascular events, through progressive endothelial damage and inflammatory cellular microenvironment [2, 5–7]. Therefore, it is important to detect in its early stages and manage cases of MetS to avoid the development of complications.

There are several international criteria to diagnose MetS, but the progress in metabolic research reveals that the environment where MetS develops is more complex than previously thought. In this regard, the interactions of endocrine and paracrine secretory products in key tissues of metabolic control are affected [4, 8]. Some of these metabolites, such as cytokines, miRNA, microvesicles/exosomes, and the components of the renin-angiotensin-aldosterone system are of particular interest as biomarkers and drug targets in MetS [9–11].

In recent years, the C-terminal sequence of pre-pro vasopressin (Copeptin), a 39‐amino acid‐long glycosylated peptide secreted equimolarly with arginine-vasopressin (AVP), has been used as an alternative marker of AVP because of its long-term stability and being easy to measure on blood [12, 13]. Copeptin is related to several cardiometabolic disorders, such as heart failure, T2DM, polycystic ovary syndrome, preeclampsia, and renal disease [14–19]. This is attributed to the overstimulation of vasopressin receptors (rV) located in different tissues involved in metabolic control [20]. In this sense, animal studies show that the convergence in rV1a and rV1b stimuli generates insulin resistance and hyperglycemia, caused by excessive activation of *β*-oxidation and adrenocorticotropic hormone release; while the coordinate activation of rV1a, rV1b, and rV2 adds water and sodium retention that induces the development of hypertension [21]. Copeptin might play an important role in MetS physiopathology and could potentially be used as an early biomarker. However, there is not enough evidence about the association between copeptin and MetS. Thus, we aim to determine the relationship between serum copeptin levels and their association with MetS in human populations because of its importance as a possible early biomarker of cardiovascular disorders related to MetS.

## 2. Methods

We performed a systematic review to assess the association between serum copeptin levels and MetS following the Preferred Reporting Items for Systematic Reviews and Meta-Analysis (PRISMA) guidelines 2021 [22] (see Supplementary Material 1). The study protocol is registered in PROSPERO (CRD42021236587).

### 2.1. Study Selection

We included original cohort, cross-sectional, and case-control studies that reported the following effect measures: risk ratio, odds ratio, hazard ratio, prevalence ratio, or data that allows estimation of any of the above-mentioned effect measures contrasting the risk of having MetS in patients with elevated copeptin levels compared to patients without elevated copeptin levels, as defined by the individual studies. Studies reporting any diagnostic criteria for MetS (National Cholesterol Education Program—Adult Treatment Panel III (ATP-III), International Diabetes Federation (IDF), American Heart Association/National Heart, Lung, and Blood Institute, and others) were included. On the other hand, we excluded case reports, editorials, commentaries, clinical practice guidelines, opinions, reviews, systematic reviews studies, and studies for which full text was not available. There were no restrictions on language or publication date.

### 2.2. Literature Search

We searched articles in four databases: (1) PubMed, (2) Web of Science, (3) Scopus, and (4) EMBASE until March 2021 (see Supplementary Material 2). Duplicated records were manually removed using the Rayyan software [23], and two review authors (BGY and RRH) independently screened the results to identify potentially relevant studies for inclusion (first reading the titles and abstracts, and after that reading the full text of the articles). Any disagreement on the selection was discussed with a third party (DRSM) and resolved by consensus. After that, we complemented the search by reviewing the lists of references of all included studies. If the full-text was not available, we sent an e-mail to the author to request the article.

### 2.3. Data Extraction

Two independent authors (BGY and RRH) independently performed data extraction from each included study using a standardized Microsoft Excel sheet, with any differences resolved by a third researcher (DRSM).

The following variables were extracted from each study: first author, year of publication, country, study design, population characteristics (number of participants, age, and sex), copeptin measurement, copeptin values, MetS diagnostic criteria, MetS prevalence, cut-off points of copeptin values, and the effective measures of the relationship between copeptin and MetS. When there were doubts about any information reported in the studies, we sent an e-mail to the authors to clarify the information.

### 2.4. Risk of Bias

We assessed the risk of bias of the included studies using the Newcastle-Ottawa Scale (NOS) [24]. To homogenize the assessments, we held training on the use of the tool and used a list of criteria to assess each of the NOS questions. The NOS has specific versions according to the study design (cross-sectional, cohort, and case-control) and consists of three domains: selection, comparability, and outcome/exposure. The maximum score for cross-sectional studies is 10, while for cohort and case-control studies is 9. Two researchers (BGY and RRH) carried out this process independently. In case of disagreement, a consensus was achieved with a third researcher (DRSM and JHZT).

### 2.5. Statistical Analyses

We present a description of the included studies and their results. We decided not to conduct meta-analyses because of the heterogeneity between the studies, different study designs, diagnostic criteria for MetS, and cut-off points for copeptin values.

## 3. Results

### 3.1. Studies Characteristics

**Figure 1:**
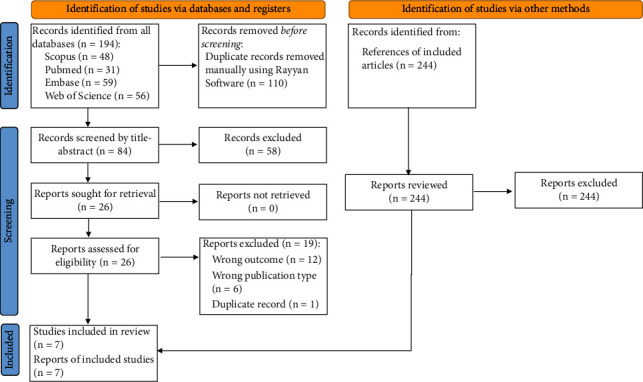
Flow diagram summarizing the process of the literature search and selection.

In the database systematic search, we identified 84 records after removing duplicates. From these, we reviewed 26 full-text for eligibility, and finally, 7 studies were included [25–31] (Supplementary Material 3). No new article was identified by reviewing the references of all included studies (Figure 1**)**.

The characteristics of the 7 studies are summarized in Table 1. The number of participants ranged from 80 to 4742. Five studies were cross-sectional, one cohort, and one case-control. Enhörning-2011 [26] and Enhörning–2013 [27] share a part of the population but have different study designs. Saleem-2009 [25] evaluated adult African-Americans (mean age 63.6 years) and non-Hispanic Whites (mean age 58.9 years) patients recruited from medical centers with high risk of cardiovascular disease, and Deligözoğlu-2020 [31] included obese children recruited in outpatient clinics, who were between 10 and 18 years of age. The rest of the studies were performed in general adult community populations with mean ages ranging from 47.4 to 57.5 years [28–30].

Concerning copeptin, the mean values ranged from 4.2 to 10.3 pmol/L, excepting in the Ertan-2020 study, where copeptin levels were much higher (30.2 pmol/L). For assessing copeptin as the exposure, the studies divided copeptin into quartiles, excepting two which divided copeptin as high and low, one based in the median [30] and the other did not report how it was divided [29].

Regarding MetS diagnosis, most of the studies used the ATP-III criteria [25–27, 29, 30] and two studies used the IDF criteria [28, 31]. The prevalence of MetS in adults found in the cross-sectional and the baseline of the cohort study ranged from 13.4 to 50.4% [25, 30], and the prevalence in the study in children with obesity was 23.8% [31].

### 3.2. Relationship between Copeptin and Metabolic Syndrome

Table 2 summarizes the relationship between copeptin and MetS. The Saleem-2009 [25] cross-sectional study conducted in the USA, assessed the association of copeptin (quartiles) and MetS in the adult general population expressed by ethnicity (African-Americans and non-Hispanic Whites). They found that the copeptin levels in both the third and fourth quartile compared with the first quartile were associated with MetS in African-Americans and non-Hispanic Whites. Likewise, the Enhorning-2011 [26] cross-sectional study conducted in the general population of Sweden, found that having higher copeptin levels (quartiles) was associated significantly with MetS. Also, they found that copeptin was associated with waist circumference, diabetes, and hyperinsulinemia. In addition, the Vintilă-2016 [29] case-control study conducted in Romania found a significant relationship between high copeptin levels (high vs. low) and MetS. However, the other studies including Enhorning-2013 [27], a cohort study, found no relationship between both the variables.

### 3.3. Risk of Bias

Regarding the risk of bias, all cross-sectional studies met the majority of items and no study gave details about the nonrespondents. The Deligözoğlu-2020 [31] study had several additional limitations such as the lack of the representativeness of the sample, an inadequate sample size, and did not adjust for confounding factors. The cohort study had no methodological issues [27]. On the other hand, the case-control study misrepresented cases, had a low response rate, and did not adjust for confounding factors. The results were summarized in Table 1 and detailed in Supplementary material 4.

## 4. Discussion

We conducted a systematic review to assess the relationship between copeptin levels and MetS and included seven studies that evaluated this relationship. However, the results of the studies were inconsistent and the association was only observed in some cross-sectional studies and one case-control study. A meta-analysis could not be performed due to the heterogeneity of the designs used, diagnostic criteria for MetS, and cut-off points for the classification of copeptin levels.

### 4.1. Association between Copeptin and MetS

The AVP regulatory system has recently been emphasized to be involved in human metabolic control, several studies consider that higher levels of copeptin are related to metabolic disorders. In this sense, the study of Saleem et al. found an association between copeptin, MetS, body mass index, plasma glucose, insulin resistance, triglycerides, and HDL-cholesterol in an American population stratified by ethnicity (African-Americans and non-Hispanic Whites) [25]. Other studies by Enhörning et al. and Vintilă et al. showed that higher quartiles or levels of copeptin were associated with MetS and its components [26, 29]. Likewise, a population-based observational study found that higher levels of copeptin were significantly related to the components of MetS, HbA1c, body mass index, water intake, and urine osmolarity [32]. In addition, some studies found that metabolic changes related to the elevated copeptin levels may also affect the liver tissue (NALF/NASH), as well as being related to MetS and insulin resistance [33, 34].

These observational studies show the existence of an association between copeptin, MetS, and metabolic disorders. However, we found a controversy in the association of copeptin and MetS. On the one hand, some studies demonstrated a directly proportional association of copeptin quartiles or levels with MetS [25, 26, 29], while other cohort and cross-sectional studies found no significant differences between the groups [27, 28, 30, 31]. In this sense, the longitudinal study by Enhörning et al. found an independent association of the copeptin levels with abdominal obesity and T2DM, two key components of MetS; however, after adjusted analysis copeptin was shown not to be associated with MetS. The authors suggest that the association of copeptin and MetS found in their previous cross-sectional study was probably driven by the association of copeptin with T2DM and abdominal obesity, core components of MetS [26, 27]. Similarly, the population-based study by Then et al. found that copeptin was associated with T2DM only in men and hypertension was associated with copeptin only in women, whereas copeptin was not associated with MetS in both the sexes [28]. The multicenter study by Canivell et al. found that copeptin was associated with insulin resistance and T2DM but not with MetS after full adjustment, additionally suggested that the age and 11*β*-HSD2 activity could modulate the association of copeptin found in this population [30]. Finally, the study of Deligözoğlu et al. showed that in obese children aged 10–18 years, copeptin was associated with masked hypertension but not with MetS [31].

Discrepancies between results and association of copeptin to MetS may be due to methodological differences. However, in most studies, copeptin was independently associated with insulin resistance, obesity, and T2DM in adjusted models, which evidences its contribution, as an indirect marker of the AVP regulatory system in metabolic disorders and key components of MetS in different populations.

### 4.2. Limitations of the Included Studies

The included studies had several limitations. It should be noted that 6 of the 7 studies assessed were cross-sectional or case-control studies, so causality could not be obtained. Of the 5 cross-sectional studies, most met the items assessed in the scale, except the Deligözoğlu-2020 study [31]. The case-control study had weaknesses in the three domains of selection, comparability, and exposure [29]. On the other hand, the cohort study had no methodological weaknesses [27]. Few studies conducted subgroup analyses and none conducted analyses of mediating factors to explain possible pathways mediating the association. Furthermore, it is important to mention that the studies were heterogeneous in methodology. As an example, studies do not present a standardized cut-off point to divide patients with normal or altered copeptin, but present quartiles, which vary from study-to-study. This renders meta-analysis an inappropriate technique to summarize the results found in the individual studies.

## 5. Implications and Recommendations

The role of copeptin as an early diagnosis surrogate marker of acute coronary syndrome and as a prognostic factor for acute myocardial infarction has been observed [35], as well as its predictive value for assessing the risk of mortality in heart failure [36, 37]. It has also been reported to be involved in the pathogenesis of diabetes mellitus [28] and its micro and macrovascular complications [38]. The pathogenesis pathways of the aforementioned associations are interrelated with the pathophysiological pathways of MetS, which in turn increases the risk of cardiovascular events [28, 35, 36, 38]. Thus, strategies based on routine copeptin measurements could help to detect early metabolic disorders in progress related to AVP pathway disorders. In this way, dietary and lifestyle interventions such as increasing water consumption [39] and reducing smoking and alcohol intake [40] would prevent the negative impact of morbidity and mortality of metabolic disorders, reduce the cost of diagnostic and therapeutic procedures, avoid prolonged monitoring, and improve the flow of medical care.

Future studies should consider standardizing bioanalysis methods and cut-off points for copeptin, to achieve this, copeptin values between 1.70 and 11.25 pmol/L could be considered as normal values since these were observed in healthy volunteers in previous studies [41]. Studies should adjust for confounding factors such as age, sex, renal, liver or heart disease, drug use, alcohol consumption, and water intake [40, 42]. In addition, subgroup analysis and the analysis of mediating factors could help to better understand the influence of copeptin on MetS.

### 5.1. Limitations and Strengths

In this review, we did not perform a meta-analysis due to heterogeneity among studies. However, a comprehensive search strategy was conducted with no language or publication date restrictions and all processes were performed in duplicate to reduce errors.

## 6. Conclusions

The association and causal relationship between copeptin and MetS are inconsistent. Some cross-sectional and case-control studies show an association, while others find no difference, including the one included in the cohort study. However, arginine-vasopressin system impairment contributes to metabolic disorders, expressing plasma copeptin changes. Thus, more longitudinal studies are required to corroborate the association of copeptin and MetS.

## Figures and Tables

**Table 1:** Characteristics of included studies assessing the relationship between copeptin and metabolic syndrome (*n* = 7).

Study id	Countries	Study designs	Sample sizes	Age (years) mean ± SD	Male sex (%)	Copeptin values (pmol/L) mean ± SD	MetS diagnostic criteria	% MetS	Quality score
Saleem-2009 (AA) [25]	USA	Cross-sectional	1293	63.6 ± 9.3	28.8	8.6 ± 5.7^a^	ATP-III	50.4	9/10
Saleem-2009 (NHW) [25]	USA	Cross-sectional	1197	58.9 ± 10.2	42.6	5.4 ± 3.4^b^	ATP-III	48.8	9/10
Enhörning-2011 [26]	Sweden	Cross-sectional	4742	57.5 ± 5.9	40.5	5.5 ± 3.7^b^	ATP-III	21.5	8/10
Enhörning-2013 [27]	Sweden	Cohort	1653	NR	40.9	NR^b^	ATP-III	26.2	9/9
Then-2015 (men) [28]	Germany	Cross-sectional	752	57.4 ± 13	100	10.3 ± 4.8^c^	IDF	41.2	9/10
Then-2015 (women) [28]	Germany	Cross-sectional	788	56.2 ± 12.7	0	7.6 ± 4^c^	IDF	25.0	9/10
Vintilă-2016 [29]	Romania	Case control	105	50.9 ± 1.4	21.9	NR^c^	ATP-III	60.0	5/9
Canivell-2017 [30]	Switzerland	Cross-sectional	1089	47.4 ± 21.5	47	4.2 ± 2.4^c^	ATP-III	13.4	8/10
Deligözoğlu-2020 [31]	Turkey	Cross-sectional	80	13.8 ± 1.93	44	30.2 ± 18.0^c^	IDF	23.8	4/10

AA: African-Americans, NHW: non-Hispanic Whites, MetS: metabolic syndrome, ATP III: adult treatment panel III, IDF: international diabetes federation, SD: standard deviation, NR: not reported, ILMA: immunoluminometric assay, CLIA: chemiluminescence immunoassay, ELISA: enzyme-linked immunosorbent assay.

**Table 2:** Relationship between copeptin and metabolic syndrome.

Study id	Copeptin categories values	MetS and copeptin (high vs. low)	Mets and copeptin (Q2 vs. Q1)	Mets and copeptin (Q3 vs. Q1)	Mets and copeptin (Q4 vs. Q1)	Adjusted variables
		Odds ratio 95% confidence interval				
Saleem-2009 (AA)	Q1 (<5.0)	NE	**1.42 (1.05–1.93)**	**1.49 (1.07–2.06)**	**2.07 (1.45–2.95)**	Age, sex, creatinine, smoking,statin or diuretic use, history of myocardial infarction/stroke, physical activity, and educational status
	Q2 (5.0–8.0)					
	Q3 (8.0–12.7)					
	Q4 (>12.7)					
Saleem-2009 (NHW)	Q1 (<3.3)	NE	1.12 (0.79–1.59)	**1.79 (1.27–2.51)**	**1.74 (1.21–2.50)**	
	Q2 (3.3–5.0)					
	Q3 (5.0–7.9)					
	Q4 (>7.9)					

Enhörning-2011	Men:	NE	**1.55 (1.25–1.93)**	**1.82 (1.47–2.25)**	**1.93 (1.57–2.39)**	None
	Q1 (<4.6)					
	Q2 (4.6–7.1)					
	Q3 (7.1–10.6)					
	Q4 (10.7–4.3)					
	Women:					
	Q1 (<2.7)					
	Q2 (2.7–4.2)					
	Q3 (4.3–6.4)					
	Q4 (6.5–14.3)					

Enhörning-2013	NR	NE	1.21 (0.85–1.72)	1.05 (0.74–1.49)	1.34 (0.95–1.91)	Follow-up time, age, sex, cystatin C, hypertension, glucose, triglycerides, HDL, and waist circumference

Then-2015 (men)	NR	NE	NE	NE	1.13 (0.72–1.76)	Age, history of myocardial infarction/stroke, smoking, alcohol intake, and physical activity
Then-2015 (women)	NR	NE	NE	NE	1.11 (0.68–1.83)	

Vintilă-2016	Low (0.1–196.4)	**20 (3.03–131.7)**	NE	NE	NE	None
	High (196.5–455.1)					

Canivell-2017	NR	1.12 (0.74–1.69)	NE	NE	NE	Age, sex, center, socioeconomic status, intake of fruits and vegetables, physical activity, alcohol intake, smoking, testosterone, and estradiol daytime urinary excretion

Deligözoğlu-2020	Q1 (<17.0)	NE	NE	0.86 (0.22–3.28)	0.33 (0.06–1.43)	None
	Q2 (17.0–26.4)					
	Q3 (26.6–40.0)					
	Q4 (40.3–95.0)					

AA: African-Americans, NHW: non-Hispanic Whites, MetS: metabolic syndrome, SD: standard deviation, NR: not reported, and NE: not evaluated. Significative values are in bold.

## Data Availability

All the data supporting the results can be found in the manuscript and supplementary material.

## Supplementary Materials

Supplementary Materials Supplementary material 1: PRISMA checklist. Supplementary material 2: search strategy by each database (Pubmed, Scopus, WoS, and Embase). Supplementary material 3: studies excluded at full-text and their reasons for exclusion. Supplementary material 4: the risk of bias of cross-sectional, cohort and case-control studies using the Newcastle-Ottawa Scale.
