# Supplementary material for: Association between Copeptin and Metabolic Syndrome: A Systematic Review
Source: J Nutr Metab. 2022 Oct 22;2022:5237903. doi: 10.1155/2022/5237903 (PMC9617695; doi:10.1155/2022/5237903)
Supplement: Supplementary Materials — Supplementary material 1: PRISMA checklist. Supplementary material 2: search strategy by each database (Pubmed, Scopus, WoS, and Embase). Supplementary material 3: studies excluded at full-text and their reasons for exclusion. Supplementary material 4: the risk of bias of cross-sectional, cohort and case-control studies using the Newcastle-Ottawa Scale. [file 5237903.f1.docx]

# Supplementary material

**Supplementary material 1. PRISMA checklist.**

| **Section and Topic** | **Item #** | **Checklist item** | **Location where item is reported** |
| --- | --- | --- | --- |
| **TITLE** | | |  |
| Title | 1 | Identify the report as a systematic review. | 1 |
| **ABSTRACT** | | |  |
| Abstract | 2 | See the PRISMA 2020 for Abstracts checklist. | 2 |
| **INTRODUCTION** | | |  |
| Rationale | 3 | Describe the rationale for the review in the context of existing knowledge. | 3 |
| Objectives | 4 | Provide an explicit statement of the objective(s) or question(s) the review addresses. | 3 |
| **METHODS** | | |  |
| Eligibility criteria | 5 | Specify the inclusion and exclusion criteria for the review and how studies were grouped for the syntheses. | 3,4 |
| Information sources | 6 | Specify all databases, registers, websites, organisations, reference lists and other sources searched or consulted to identify studies. Specify the date when each source was last searched or consulted. | 4 |
| Search strategy | 7 | Present the full search strategies for all databases, registers and websites, including any filters and limits used. | 4, S2 |
| Selection process | 8 | Specify the methods used to decide whether a study met the inclusion criteria of the review, including how many reviewers screened each record and each report retrieved, whether they worked independently, and if applicable, details of automation tools used in the process. | 4 |
| Data collection process | 9 | Specify the methods used to collect data from reports, including how many reviewers collected data from each report, whether they worked independently, any processes for obtaining or confirming data from study investigators, and if applicable, details of automation tools used in the process. | 4 |
| Data items | 10a | List and define all outcomes for which data were sought. Specify whether all results that were compatible with each outcome domain in each study were sought (e.g. for all measures, time points, analyses), and if not, the methods used to decide which results to collect. | 4 |
|  | 10b | List and define all other variables for which data were sought (e.g. participant and intervention characteristics, funding sources). Describe any assumptions made about any missing or unclear information. | 4 |
| Study risk of bias assessment | 11 | Specify the methods used to assess risk of bias in the included studies, including details of the tool(s) used, how many reviewers assessed each study and whether they worked independently, and if applicable, details of automation tools used in the process. | 4 |
| Effect measures | 12 | Specify for each outcome the effect measure(s) (e.g. risk ratio, mean difference) used in the synthesis or presentation of results. | - |
| Synthesis methods | 13a | Describe the processes used to decide which studies were eligible for each synthesis (e.g. tabulating the study intervention characteristics and comparing against the planned groups for each synthesis (item #5)). | 5 |
|  | 13b | Describe any methods required to prepare the data for presentation or synthesis, such as handling of missing summary statistics, or data conversions. | 5 |
|  | 13c | Describe any methods used to tabulate or visually display results of individual studies and syntheses. | 5 |
|  | 13d | Describe any methods used to synthesize results and provide a rationale for the choice(s). If meta-analysis was performed, describe the model(s), method(s) to identify the presence and extent of statistical heterogeneity, and software package(s) used. | 5 |
|  | 13e | Describe any methods used to explore possible causes of heterogeneity among study results (e.g. subgroup analysis, meta-regression). | - |
|  | 13f | Describe any sensitivity analyses conducted to assess robustness of the synthesized results. | - |
| Reporting bias assessment | 14 | Describe any methods used to assess risk of bias due to missing results in a synthesis (arising from reporting biases). | - |
| Certainty assessment | 15 | Describe any methods used to assess certainty (or confidence) in the body of evidence for an outcome. | - |
| **RESULTS** | | |  |
| Study selection | 16a | Describe the results of the search and selection process, from the number of records identified in the search to the number of studies included in the review, ideally using a flow diagram. | 5, F1 |
|  | 16b | Cite studies that might appear to meet the inclusion criteria, but which were excluded, and explain why they were excluded. | S3 |
| Study characteristics | 17 | Cite each included study and present its characteristics. | T1 |
| Risk of bias in studies | 18 | Present assessments of risk of bias for each included study. | T1, S4 |
| Results of individual studies | 19 | For all outcomes, present, for each study: (a) summary statistics for each group (where appropriate) and (b) an effect estimate and its precision (e.g. confidence/credible interval), ideally using structured tables or plots. | T2 |
| Results of syntheses | 20a | For each synthesis, briefly summarise the characteristics and risk of bias among contributing studies. | - |
|  | 20b | Present results of all statistical syntheses conducted. If meta-analysis was done, present for each the summary estimate and its precision (e.g. confidence/credible interval) and measures of statistical heterogeneity. If comparing groups, describe the direction of the effect. | - |
|  | 20c | Present results of all investigations of possible causes of heterogeneity among study results. | - |
|  | 20d | Present results of all sensitivity analyses conducted to assess the robustness of the synthesized results. | - |
| Reporting biases | 21 | Present assessments of risk of bias due to missing results (arising from reporting biases) for each synthesis assessed. | - |
| Certainty of evidence | 22 | Present assessments of certainty (or confidence) in the body of evidence for each outcome assessed. | - |
| **DISCUSSION** | | |  |
| Discussion | 23a | Provide a general interpretation of the results in the context of other evidence. | 8,9 |
|  | 23b | Discuss any limitations of the evidence included in the review. | 9 |
|  | 23c | Discuss any limitations of the review processes used. | 9 |
|  | 23d | Discuss implications of the results for practice, policy, and future research. | 9 |
| **OTHER INFORMATION** | | |  |
| Registration and protocol | 24a | Provide registration information for the review, including register name and registration number, or state that the review was not registered. | 3 |
|  | 24b | Indicate where the review protocol can be accessed, or state that a protocol was not prepared. | 3 |
|  | 24c | Describe and explain any amendments to information provided at registration or in the protocol. | - |
| Support | 25 | Describe sources of financial or non-financial support for the review, and the role of the funders or sponsors in the review. | 10 |
| Competing interests | 26 | Declare any competing interests of review authors. | 10 |
| Availability of data, code and other materials | 27 | Report which of the following are publicly available and where they can be found: template data collection forms; data extracted from included studies; data used for all analyses; analytic code; any other materials used in the review. | - |

## Supplementary material 2. Search strategy

| Database | Search strategy | Date of Search | Results |
| --- | --- | --- | --- |
| Pubmed | #1 Metabolic syndrome  "Metabolic Syndrome"[Mesh] OR "Metabolic Syndrom*"[tiab] OR “Insulin Resistance Syndrome”[tiab] OR “Dysmetabolic Syndrome X”[tiab] OR “Reaven Syndrome X”[tiab] OR “Metabolic Cardiovascular Syndrome”[tiab] OR “Cardiometabolic Syndrom*”[tiab] OR "Metabolic Syndrom*"[OT] OR “Insulin Resistance Syndrome” [OT] OR “Dysmetabolic Syndrome X”[OT] OR “Reaven Syndrome X” [OT] OR “Metabolic Cardiovascular Syndrome” [OT] OR “Cardiometabolic Syndrom*”[OT]  #2 Copeptin  Copeptins [Supplementary Concept] OR Copeptin* [TIAB] OR ((C-terminal [TIAB] OR “C terminal” [TIAB]) AND (AVP [TIAB] OR vasopressin [TIAB] OR proargipressin [TIAB] OR provasopressin [TIAB])) OR Copeptin* [OT] OR ((C-terminal [OT] OR “C terminal” [OT]) AND (AVP [OT] OR vasopressin [OT] OR proargipressin [OT] OR provasopressin [OT]))  (#1 AND #2) | 16/03/2021 | 31 |
| Scopus | TITLE-ABS-KEY(“Insulin Resistance Syndrome” OR “Reaven Syndrome X”) OR TITLE-ABS-KEY (*metabolic W/2 Syndrom*)  TITLE-ABS-KEY(Copeptin* OR ((C-terminal OR “C terminal”) W/3 (AVP OR vasopressin OR proargipressin OR provasopressin)))  (#1 AND #2) | 16/03/2021 | 48 |
| WOS | TS=(“Insulin Resistance Syndrome” OR “Reaven Syndrome X”) OR TS=(*metabolic NEAR/2 Syndrom*)  TS=(Copeptin* OR ((C-terminal OR “C terminal”) NEAR/3 (AVP OR vasopressin OR proargipressin OR provasopressin)))  (#1 AND #2) | 16/03/2021 | 56 |
| Embase | ‘metabolic syndrome X’/exp OR (‘Insulin Resistance Syndrome’ OR ‘Reaven Syndrome X’):ti,ab,kw OR ((metabolic OR dysmetabolic OR cardiometabolic) NEAR/2 Syndrom*):ti,ab,kw  Copeptin/exp OR (Copeptin* OR ((C-terminal OR ‘C terminal’) NEAR/3 (AVP OR vasopressin OR proargipressin OR provasopressin))):ti,ab,kw  (#1 AND #2) | 16/03/2021 | 59 |

## Supplementary material 3. Excluded studies.

| Author - Year | Title | Reason for exclusion |
| --- | --- | --- |
| Tuli - 2021 | Distribution of plasma copeptin levels and influence of obesity in children and adolescents | wrong outcome |
| Brunkwall - 2020 | High water intake and low urine osmolality are associated with favorable metabolic profile at a population level: low vasopressin secretion as a possible explanation | wrong outcome |
| Vasileva - 2018 | Should we measure copeptin levels in patients with pre-metabolic and metabolic syndrome? | wrong outcome |
| Rothermel - 2016 | Copeptin in obese children and adolescents: relationships to body mass index, cortisol and gender | wrong outcome |
| Roussel - 2016 | Plasma copeptin, AVP gene variants, and incidence of type 2 diabetes in a cohort from the community | wrong outcome |
| Tenderenda-Banasiuk - 2014 | Serum copeptin levels in adolescents with primary hypertension | wrong outcome |
| Eltabakh - 2018 | Copeptin as a novel biomarker of obesity-induced insulin resistance and metabolic syndrome | wrong publication type |
| Jensen - 2017 | Copeptin, waist circumference, and insulin sensitivity in type 1 diabetes | wrong publication type |
| Canivell - 2015 | Plasma copeptin is associated with insulin resistance in a SWISS population-based study | wrong publication type |
| Baranowska - 2014 | The diagnostic significance of copeptin | wrong publication type |
| Tenderenda-Banasiuk - 2014 | Serum copeptin levels in adolescents with primary hypertension | wrong publication type |
| Doerr - 2012 | Copeptin and adrenomedullin in a large cohort of catheterisation laboratory patients with newly detected diabetes or impaired glucose tolerance: The Silent Diabetes Study | wrong publication type |
| Kusche - 2012 | Copeptin and adrenomedullin in a large cohort of patients with coronary heart disease and newly diagnosed glucose intolerance (“Silent diabetes study”) | Duplicate |
| Emre - 2020 | Assessment of copeptin, ghrelin and pro BNP in metabolic syndrome | wrong outcome |
| Thomsen - 2019 | Association of copeptin, a surrogate marker for arginine vasopressin secretion, with insulin resistance: Influence of adolescence and psychological stress | wrong outcome |
| Taskin - 2015 | Circulating levels of obestatin and copeptin in obese and nonobese women with polycystic ovary syndrome | wrong outcome |
| Tufvesson - 2013 | Diabetes Mellitus and Elevated Copeptin Levels in Middle Age Predict Low Cognitive Speed after Long-Term Follow-Up | wrong outcome |
| Abbasi - 2012 | Sex differences in the association between plasma copeptin and incident type 2 diabetes: the Prevention of Renal and Vascular Endstage Disease (PREVEND) study | wrong outcome |
| Enhorning - 2010 | Plasma Copeptin and the Risk of Diabetes Mellitus | wrong outcome |

#

## Supplementary material 4. Risk of bias of included studies

| Risk of bias of cross-sectional studies | | | | | | | | |  |
| --- | --- | --- | --- | --- | --- | --- | --- | --- | --- |
| Study | Selection (Max. 5 *) | | | | Comparability (Max. 2*) | Outcome (Max. 3*) | | Final score (Max. 10) |  |
|  | Representativeness of the sample | Sample size | Non-respondents | Ascertainment of the exposure (risk factor) | The subjects in different outcome groups are comparable, based on the study design or analysis. Confounding factors are controlled. | Ascertainment of exposure | Statistical test |  |  |
| Saleem - 2009 | * | * | - | ** | ** | ** | * | 9 |  |
| Enhörning - 2011 | * | * | - | ** | * | ** | * | 8 |  |
| Then - 2015 | * | * | - | ** | ** | ** | * | 9 |  |
| Canivell - 2017 | * | * | - | ** | * | ** | * | 8 |  |
| Deligözoğlu - 2020 | - | - | - | * | - | ** | * | 4 |  |
| The scale is scored with a minimum score of zero and a maximum of ten stars. Each item has a maximum of one star except for the “Ascertainment of the exposure (risk factor)” and "comparability" items which can have two stars. The higher the number of stars, the lower the risk of bias. | | | | | | | | |  |

| Risk of bias of cohort study | | | | | | | | | |
| --- | --- | --- | --- | --- | --- | --- | --- | --- | --- |
|  | Selection (Max. 4*) | | | | Comparability (Max 2*) | Exposure (Max. 3*) | | |  |
| Study | Representativeness of the exposed cohort | Selection of the non exposed cohort | Ascertainment of exposure | Demonstration that outcome of interest was not present at start of study | Comparability of cohorts on the basis of the design or analysis | Assessment of outcome | Was follow-up long enough for outcomes to occur | Adequacy of follow up of cohorts | Final score (Max. 9) |
| Enhörning - 2013 | * | * | * | * | ** | * | * | * | 9 |
| The scale is scored with a minimum score of zero and a maximum of nine stars. Each item has a maximum of one star except for the "comparability" item which can have two stars. The higher the number of stars, the lower the risk of bias. | | | | | | | | | |

| Risk of bias of case-control study | | | | | | | | | |
| --- | --- | --- | --- | --- | --- | --- | --- | --- | --- |
| Study | Selection (Max. 4*) | | | | Comparability (Max 2*) | Exposure (Max. 3*) | | |  |
|  | Is the case definition adequate? | Representativeness of the cases | Selection of Controls | Definition of Controls | Comparability of cases and controls on the basis of the design or analysis | Ascertainment of exposure | Same method of ascertainment for cases and controls | Non-Response rate | Final score (Max. 9) |
| Vintilă - 2016 | * | - | * | * | - | * | * | - | 5 |
| The scale is scored with a minimum score of zero and a maximum of nine stars. Each item has a maximum of one star except for the "comparability" item which can have two stars. The higher the number of stars, the lower the risk of bias. | | | | | | | | | |
